# Food insecurity among disabled adults

**DOI:** 10.1093/eurpub/ckac034

**Published:** 2022-05-13

**Authors:** Mia Hadfield-Spoor, Mauricio Avendano, Rachel Loopstra

**Affiliations:** Department of Nutritional Sciences, King’s College London, London, UK; Department of Epidemiology and Health Systems, Center for Primary Care and Public Health (Unisanté), University of Lausanne, Lausanne, Switzerland; Department of Nutritional Sciences, King’s College London, London, UK

## Abstract

**Background:**

The relationship between disability and food insecurity is under-researched. Risk of food insecurity may vary by type and number of disabilities. We examine the hypotheses that (i) a higher number of disabilities increases risk of food insecurity and (ii) associations of physical disabilities, mental/cognitive disabilities or a combination of both types with food insecurity may differ in strength.

**Methods:**

Data came from the fifth wave of the UK’s Food Standards Agency’s Food and You survey (2018), which contains detailed information on disability and household food insecurity. We used logistic and multinomial logistic regression to model the number and type of disabilities as predictors for food insecurity outcomes, controlling for socio-demographic factors.

**Results:**

Both type and number of disabilities predicted food insecurity. Every additional disability was associated with higher odds of food insecurity [odds ratio (OR): 1.60, 95% confidence interval (CI): 1.40–1.83]. Among people with a disability, every additional disability was associated with 19% higher odds of food insecurity (OR: 1.19, 95% CI: 1.05–1.34). People with both physical and mental/cognitive disabilities had increased odds of severe food insecurity (OR: 8.97, 95% CI: 3.54–22.7).

**Conclusion:**

Number and type of disabilities are associated with higher risk of food insecurity. A combination of physical and mental/cognitive disabilities, as well as having multiple disabilities are each independently associated with higher risk of food insecurity. Policy-makers may thus consider using targeted and tailored policies to reduce barriers to social and financial inclusion of disabled people to reduce food insecurity.

## Introduction

An emerging literature on food insecurity and disability has shown that disability is associated with higher risk of food insecurity.^1–3^ According to the biopsychosocial model of disability,^4^ disability is understood as an interaction between a person and social context. Thus, the relationship between disability and food insecurity may reflect the fact that disabled people are at higher risk of socio-economic disadvantage and exclusion^5^^,^^6^ due to facing significant barriers to education, work, adequate income and financial security.^7^^,^^8^^,^^9^ Disabled people also endure higher costs of living and are more likely to experience ill-health.^10^^,^^11^ Lower socio-economic status and ill-health have both been shown to increase the risk of food insecurity.^12^^,^^13^

While studies in high-income countries have found that food insecurity is strongly associated with mental,^2^^,^^14^^,^^15^ physical and chronic illness,^16^^,^^17^^,^^18^ research looking at the relationship between disability and food insecurity is limited and of mixed quality.^3^ This relationship is likely to be bidirectional, as food insecurity may increase the risk of physical disability, while at the same time, poor health among disabled people may lead to food insecurity.^19^^,^^20^ A limited number of studies in high-income countries suggest that the type and intensity of disability, as well as chronicity of food insecurity, may be important to understand the relationship between disability and food insecurity. Previous studies have focused primarily on the USA or have faced important limitations regarding the measurement and modelling of food insecurity. Studies in the USA and Canada have found that work-limiting disabilities are associated with food insecurity, as well as being disabled and of working age.^2^^,^^17^^,^^21^ Functional disabilities such as mobility limitations, barriers particularly faced by physically disabled people, as well as barriers as a result of cognitive impairments, have been associated with a higher risk of food insecurity.^22^^,^^23^ Studies also suggest that some groups of disabled people, such as people with lower or more insecure incomes, may be at higher risk of facing food insecurity.^12^^,^^24^ However, few studies have examined how different types of disability as well as the number of disabilities relate to the risk of food insecurity, particularly in the UK context. A better understanding of how different experiences of disability relate to food insecurity and to what extent food insecurity risk varies among disabled people is critical for developing targeted and tailored policies and programmes for reducing food insecurity among disabled people.

In this paper, we explore the hypothesis that categories and number of disabilities are independently associated with food insecurity. This hypothesis is motivated by literature suggesting that different types of disability pose different barriers and facilitators to inclusion and equality of access to adequate food. For example, mentally/cognitively disabled people may face particular knowledge, information and income and work barriers, such as difficulty building or maintaining social networks and facing discrimination and stigma.^25^ On the other hand, physically disabled people may be more likely to face particular food access barriers associated with the physical and built environment.^26–28^ Based on this literature, we focus on two hypotheses. Firstly, we hypothesize that having multiple disabilities puts people at higher risk of food insecurity due to more and/or a higher intensity of barriers to access and participation that increase social disadvantage,^29^ e.g. public transport and supermarket access, exclusion from secure and sufficient income, higher likelihood of poorer health.^30^ Second, we hypothesize that mentally/cognitively disabled people have higher risk of food insecurity than people who are physically disabled due to lack of parity in terms of social support and support services which could lead to higher unmet need^31^ and that people with a combination of categories will experience higher risk of food insecurity than people who have only physical or only mental disabilities.

## Methods

### Data source and sample

Data came from the UK’s Food Standards Agency’s Food & You survey (F&Y), a repeated cross-sectional, representative survey of adults aged 16 and over in England, Wales and Northern Ireland.^32^ The survey uses random probability sampling and face-to-face computer-assisted personal interviewing. At the time of analysis, it was the only nationally representative dataset in the UK containing an internationally agreed measure of household food insecurity: the United States Department of Agriculture (USDA)’s Adult Food Security Survey Module.^33^ Data from Wave 5 of F&Y, conducted in 2018, were used, as this wave collected detailed information about disability.^32^ Of the 6346 eligible addresses approached, the response rate was 48.2%, resulting in a sample size of 3059 adults.^32^

### Number and type of disability

**Figure 1:**
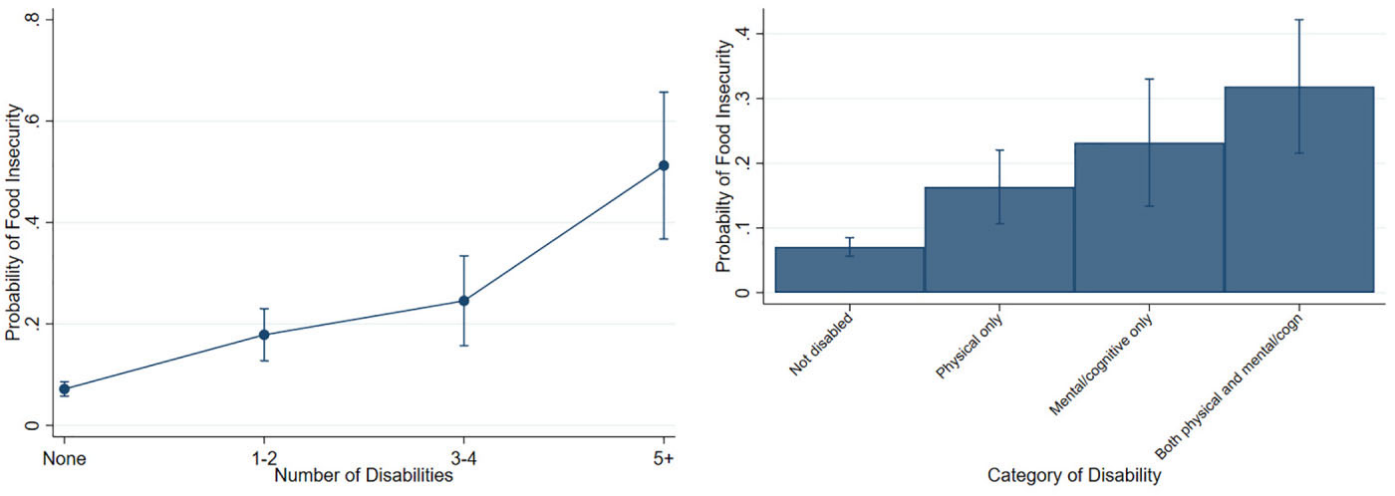
Relationship of food insecurity with number and category of disabilities. Probability of food insecurity by disability number and category. *Note:* Predicted probabilities adjusted for age

In line with the definition of disability in the UK Equality Act,^34^ respondents were asked if they had any physical or mental health conditions or illnesses lasting or expected to last for 12 months or more. Respondents who answered affirmatively were then asked whether any conditions or illnesses affected them in any of nine specific areas: mental health; social or behavioural problems; memory; learning, understanding or concentration problems; vision; hearing; mobility; dexterity; or stamina or breathing or fatigue. Respondents could indicate if their condition affected them in other areas or ‘none of the above’. Based on this information, we constructed a continuous variable that indicated the number of areas of disability, which ranged from 0 to 8. To provide visualization of the age-adjusted relationship between number of disability areas and food insecurity (figure 1), we also created a four-level categorical variable (0: no disabilities; 1–2, 3–4 and 5 or more).

We created a separate categorical variable that captured the broad type of disability individuals experienced. Subcategories were as follows: no disability; physical (vision, hearing, mobility, dexterity, stamina/breathing/fatigue) disability only; mental or cognitive (social/behavioural, memory, learning, understanding/concentration) disability only; or both physical and cognitive/mental disability. We combined cognitive and mental disabilities into a single category based on prior literature (i.e. disabling mental ill-health can be considered a cognitive limitation), and we expected barriers and impacts associated with these types of disability to share common mechanisms.^2^^,^^23^ We grouped a range of physical disabilities into one category, each of which may have different associations with food insecurity.^22^ Unfortunately, low sample sizes for each individual physical condition precluded a more refined analysis for specific disabilities.

People who indicated having physical or mental health conditions or illnesses but who did not provide information on domains affected by them (i.e. selected ‘other’) were excluded (*n* = 124), as it was not possible to accurately establish the number of areas in which disability was experienced.

### Food insecurity

Food insecurity was measured by the USDA’s 10-item Adult Food Security module, a validated scale that aims to capture the prevalence of food insecurity in the general population. According to standard USDA practice, food insecurity is identified by three or more affirmative responses to questions on the module, and severe food insecurity is identified by six or more affirmative responses. At this level, respondents have indicated experiences of going without food. In addition to examining food insecurity and severe food insecurity outcomes, a measure of chronicity of food insecurity was derived from the first three module questions which ask respondents how often they worried about running out of food; how often food actually ran out; and how often they could not afford to eat balanced meals. Respondents who indicated ‘Never true’ for all three questions were coded as not experiencing food insecurity; respondents who indicated ‘sometimes true’ to at least one question, but did not indicate ‘often’ in any of the questions, were coded as ‘sometimes experiencing food insecurity’. Finally, respondents who indicated ‘often true’ to at least one question were coded as ‘often experiencing food insecurity’. Ten respondents were excluded as they provided no information on these questions.

### Control variables

Reflecting a biopsychosocial model of disability, we control for socio-economic factors that may influence experiences of disability. The following control variables were used: age group (16–24, 25–34, 35–44, 45–54, 55–64, 65–74, 75+), gender, ethnicity (white vs ‘other’), household composition (single and no children, single with children, married with no children and married with children); work status (in work, retired, unemployed, ‘other’), income bracket (<£10 399, £10 400–£25 999, £26 000–51 999, >£52 000, missing) and level of education (no qualification, ‘other’, university degree); ‘other’ referred to another kind of educational, professional, vocational or work-related qualification. Household income was not reported by 754 respondents (24.6%) so a derived variable was created with a new level for ‘missing’. The numbers of respondents missing data for other covariates were as follows: age (*n* = 9, 0.29%), sex (*n* = 0, 0.00%), highest qualification (*n* = 16, 0.52%), work status (*n* = 1, 0.03%), household composition (*n* = 11, 0.36%) and ethnicity (*n* = 13, 0.42%). Across these covariates combined, 16 respondents were dropped from the analysis due to missing data.

### Statistical analysis

Logistic regression was used to model the probability of ‘any’ and ‘severe’ food insecurity as a function of (i) the number of disabilities and (ii) the type of disability, controlling for socio-demographic variables. In a third model, we examine if, conditional on having at least one disability, the number of disabilities is associated with increased odds of food insecurity. This model captures the risk associated with each additional disability among those who have multiple disabilities. We use the same modelling strategy but a multinomial regression model to examine the chronicity of food insecurity as outcome variable.

### Sensitivity analyses

Relationships between disability and food insecurity may be stronger among younger people. Disability becomes more prevalent in older ages, affecting a wider range of socio-demographic groups. In addition, older people may become eligible for pensions or other welfare programmes, which reduces their risk of food insecurity. These factors may mean that disability is less strongly associated with food insecurity at older ages. To examine this, we present results stratified by age, using two alternative cut-offs: age 55 (the age at which claims for disability benefits start to increase rapidly) and age 65 (a common age cut-off used to define older age in the ageing literature).

All analyses use survey weights provided in the F&Y data to account for sampling design and stratification.

## Results

### Descriptive statistics

Table 1 summarizes key descriptive statistics and shows that 22% of respondents reported a disability. Thirteen percent of the sample had a physical (but not mental/cognitive) disability; 4% had a mentally/cognitive (but not physical) disability, while 5% had both a physical and mental/cognitive disability. Higher proportions of respondents who had a mental/cognitive disability and both physical and mental/cognitive disabilities reported any, severe and more chronic food insecurity than respondents who had physical or no disabilities. There were higher proportions of people in older age groups among physically disabled people, while mental/cognitive disabilities were more concentrated at ages 18–64.

**Table 1:** Descriptive statistics for non-disabled people and by disability category (*n* = 3609)

	Not disabled		Physical only		Mental/cognitive only		Physical and mental/cognitive	
	*n*	% (95% CI)	*n*	% (95% CI)	*n*	% (95% CI)	*n*	% (95% CI)
Total								
Whole sample	2094	78.0 (75.9–80.0)	513	13.3 (11.8–15.0)	110	3.65 (2.78–4.78)	215	4.99 (4.16–5.97)
No. of disabilities								
None	2094	1	–	–	–	–	–	–
1–2	0	0	408	84.1 (79.9–87.6)	102	88.4 (75.7–94.9)	40	20.1 (13.2–29.4)
3–4	0	0	99	15.0 (11.6–19.1)	8	11.6 (5.06–24.3)	111	49.5 (39.9–59.1)
5+	0	0	6	0.94 (0.35–2.51)	0	0	64	30.4 (22.2–40.1)
Age								
16–24	160	14.8 (12.6–17.4)	10	5.58 (2.38–12.5)	10	18.3 (9.31–26.1)	4	10.7 (3.89–26.1)
25–34	344	19.0 (16.5–21.7)	15	4.35 (2.45–7.62)	24	24.3 (16.0–35.2)	19	9.12 (4.20–18.7)
35–44	373	17.3 (15.4–19.5)	30	6.48 (4.17–9.94)	27	20.2 (12.5–30.9)	24	8.10 (4.68–13.7)
45–54	380	19.1 (16.8–21.6)	49	11.8 (8.40–16.3)	22	14.2 (8.39–22.9)	41	18.9 (12.8–27.1)
55–64	323	12.8 (11.1–14.8)	107	19.9 (15.8–24.9)	17	15.5 (9.36–24.4)	50	22.2 (15.4–30.9)
65–74	305	10.8 (9.33–12.5)	146	27.0 (22.0–32.6)	8	6.95 (3.11–14.8)	31	12.3 (7.78–19.0)
75+	203	6.13 (5.10–7.36)	155	24.9 (20.3–30.1)	2	0.60 (0.11–3.39)	46	18.7 (12.5–27.0)
Sex								
Female	1214	50 (47.1–52.9)	311	54.6 (49.0–60.0)	60	49.1 (35.7–62.5)	135	54.2 (43.6–64.4)
Male	880	50 (47.1–52.9)	202	45.4 (40.0–51.0)	50	51.0 (37.5–64.3)	80	45.8 (35.7–56.4)
Work status								
In work	1262	68.5 (65.3–71.5)	123	32.6 (26.9–38.8)	59	59.0 (47.8–69.4)	50	32.5 (23.4–43.0)
Retired	517	16.5 (14.4–18.8)	307	51.5 (45.4–57.5)	10	7.56 (3.56–15.3)	81	31.9 (24.0–41.0)
Unemployed	75	2.83 (2.17–3.69)	12	3.25 (1.59–6.54)	17	12.9 (6.81–23.2)	16	10.0 (5.62–17.3)
Other	239	12.2 (10.3–14.3)	71	12.7 (10.3–14.3)	24	20.5 (12.7–31.3)	68	25.6 (17.7–35.6)
Qualification								
University degree	673	34.5 (31.6–37.5)	108	23.5 (19.0–28.8)	26	24.3 (12.8–41.2)	34	20.2 (14.0–28.3)
Other	1054	50.9 (47.7–54.1)	252	51.9 (45.6–58.2)	63	62.8 (46.6–76.6)	116	56.3 (46.8–65.3)
None	360	14.6 (12.4–17.2)	153	24.5 (19.7–30.1)	21	12.9 (7.04–22.4)	65	23.5 (17.3–31.1)
HH income								
<£10 399	159	4.34 (3.46–5.43)	59	8.08 (5.85–11.1)	18	7.51 (4.12–13.3)	33	9.76 (6.52–14.4)
£10 400–£25 999	459	16.1 (14.2–18.2)	151	26.8 (22.3–31.7)	41	33.0 (23.3–44.3)	69	25.7 (19.4–33.2)
£26 000–£51 999	541	25.6 (23.0–28.4)	115	24.2 (19.9–29.2)	12	13.6 (7.46–23.4)	32	16.5 (10.9–24.4)
>£52 000	437	25.4 (22.8–28.1)	54	14.2 (10.0–19.8)	17	20.1 (10.8–34.4)	22	11.0 (6.38–18.4)
Missing	498	28.6 (25.1–32.4)	134	26.7 (21.9–32.2)	22	25.9 (16.2–38.6)	59	37.0 (27.6–47.4)
HH composition								
Married with kids	462	25.6 (23.0–28.4)	29	9.66 (6.21–14.7)	18	17.0 (10.4–26.5)	27	16.3 (10.0–25.4)
Single with kids	157	6.27 (4.99–7.85)	25	7.22 (3.79–13.3)	7	4.67 (1.52–13.5)	17	8.51 (3.39–19.8)
Married no kids	780	39.6 (36.6–42.7)	236	54.2 (48.1–60.3)	30	26.2 (17.9–36.6)	68	37.4 (29.2–46.4)
Single no kids	689	28.5 (25.7–31.6)	220	28.9 (24.5–33.7)	55	52.1 (41.4–62.6)	102	37.8 (28.7–47.8)
Ethnicity								
White	1867	83.3 (79.6–86.5)	490	94.0 (91.0–96.0)	104	96.3 (91.3–98.5)	204	88.3 (77.2–94.4)
Not white	222	16.7 (13.6–20.5)	23	6.00 (3.97–8.97)	6	3.70 (1.52–8.71)	11	11.7 (5.60–22.8)
Food security status								
High FS	1726	82.3 (79.8–84.6)	422	80.5 (75.1–85.0)	64	60.7 (49.0–71.3)	134	59.8 (49.5–69.3)
Marginal FS	203	10.1 (8.40–12.2)	39	9.10 (6.11–13.4)	13	11.8 (6.61–20.2)	30	13.4 (8.49–20.4)
Food insecurity	99	4.83 (3.74–6.21)	33	7.58 (4.73–11.9)	16	16.4 (9.24–27.4)	14	9.69 (5.23–17.3)
Severe food insecurity	66	2.71 (1.97–3.73)	19	2.81 (1.61–4.87)	17	11.1 (5.96–19.8)	37	17.2 (10.7–26.5)
Chronicity of FI								
Never	1724	82.5 (79.9–84.7)	421	80.5 (75.0–84.9)	63	60.5 (48.6–71.2)	134	59.9 (49.6–69.4)
Sometimes	266	13.3 (11.4–15.6)	60	12.6 (9.05–17.2)	27	24.9 (16.4–36.0)	52	23.1 (16.6–31.3)
Often	100	4.24 (3.16–5.65)	31	6.99 (4.43–10.9)	18	14.6 (8.25–24.6)	28	17.0 (10.1–27.1)

Note: *P*-values for all covariates ≤0.0001 except for sex (*P* = 0.5331).

Disability was associated with several forms of social and economic disadvantage. Disabled people were less likely to be in work and more likely to be retired (if physically disabled only), unemployed or not working for other reasons (if mentally/cognitive disabled only). For people who reported combined disabilities, larger proportions were either retired or not working. Disabled people were less likely to have achieved a degree qualification and were more likely to have an annual income below £25 999.

### Food insecurity and the type and number of disabilities

Figure 1 shows predicted probabilities of food insecurity derived from a logistic regression model that controlled for age. The probability of food insecurity increased linearly with the number of disabilities. To illustrate, 51% [95% confidence interval (CI): 37–66%] of people who had five or more disabilities reported food insecurity, compared with only 7% (95% CI: 6–9%) of non-disabled people and 18% (95% CI: 13–23%) for people with 1–2 disabilities. The age-adjusted prevalence of food insecurity was 16% (95% CI: 11–22%) for people physically disabled; 23% (95% CI: 13–33%) for people mentally/cognitively disabled; and 32% (95% CI: 22–42%) for people both physically and mentally/cognitively disabled. Similar associations were observed for severe food insecurity (Supplementary figure SA1) and chronic food insecurity (Supplementary figure SA2).

Table 2 shows the results of logistic regression models that adjusted for socio-economic and demographic controls. Model 1 shows that every additional disability was associated with higher odds of food insecurity [odds ratio (OR): 1.60, 95% CI: 1.40–1.83]. Model 2 shows there was an increased risk of food insecurity for people who had a physical (OR: 2.58, 95% CI: 1.45–4.60), mental/cognitive (OR: 3.17, 95% CI: 1.85–7.47) or both physical and mental/cognitive disability (OR: 6.21, 95% CI: 3.22–12.0). Among disabled people (model 3), each additional disability conferred a 19% increased odds (OR: 1.19, 95% CI: 1.05–1.34) of food insecurity. In models that used severe food insecurity as the outcome (see Supplementary table SA1), number of disabilities and a combination of physical and mental/cognitive disability predicted severe food insecurity, but not physical or mental/cognitive disabilities on their own.

**Table 2:** Odds of food insecurity for number, category and number if disabled

	Model 1	Model 2	Model 3^a^
	Number	Category	Number if disabled
	OR (95% CI)	OR (95% CI)	OR (95% CI)
Number			
Per disability	**1.60 (1.40**–**1.83)**	–	**1.19 (1.05**–**1.34)**
Category (reference=None)			
Physical only	–	**2.58 (1.45**–**4.60)**	**–**
Mental/cognitive only	–	**3.71 (1.85**–**7.47)**	**–**
Physical and mental/cognitive	–	**6.21 (3.22**–**12.0)**	**–**
Age (reference = 45–54)			
16–24	1.67 (0.82–3.41)	1.59 (0.76–3.31)	1.13 (0.41–3.15)
25–34	**1.94 (1.10**–**3.42)**	**1.89 (1.05**–**3.41)**	1.44 (0.68–3.06)
35–44	**2.55 (1.40**–**4.65)**	**2.45 (1.31**–**4.56)**	1.52 (0.64–3.00)
55–64	0.76 (0.38–1.52)	0.79 (0.40–1.56)	0.66 (0.36–1.22)
65–74	**0.32 (0.14**–**0.75)**	**0.34 (0.15**–**0.76)**	**0.32 (0.11**–**0.88)**
75+	**0.13 (0.03**–**0.53)**	**0.16 (0.04**–**0.61)**	**0.56 (0.01**–**0.21)**
Sex (reference=male)			
Female	1.41 (0.92–2.15)	1.36 (0.88–2.10)	1.14 (0.74–1.76)
Ethnicity (reference=White)			
Other ethnicity	1.48 (0.90–2.45)	1.58 (0.94–2.68)	**3.64 (1.72**–**7.72)**
Qualification (reference=university degree)			
Other	1.53 (0.90–2.60)	1.55 (0.90–2.66)	1.52 (0.82–2.81)
None	**4.13 (2.06**–**8.29)**	**4.18 (2.04**–**8.55)**	**2.55 (1.28**–**2.11)**
Work status (reference=in work)			
Retired	0.68 (0.32–1.42)	0.66 (0.33–1.34)	1.09 (0.43–2.73)
Unemployed	**2.18 (1.11**–**4.27)**	2.01 (0.98–4.12)	**2.46 (1.14**–**4.89)**
Other	**0.59 (0.37**–**0.95)**	0.64 (0.40–1.01)	1.22 (0.71–2.11)
HH income (reference= £26 000–51 999)			
<£10 399	**2.46 (1.40**–**4.34)**	**2.49 (1.42**–**4.35)**	**3.13 (1.45**–**6.74)**
£10 400–£25 999	**2.28 (1.39**–**3.73)**	**2.18 (1.34**–**3.56)**	1.67 (0.84–3.29)
>£52 000	**0.33 (0.17**–**0.64)**	**0.33 (0.17**–**0.61)**	0.60 (0.22–1.59)
Missing	1.02 (0.61–1.73)	1.03 (0.60–1.74)	1.23 (0.57–2.63)
HH composition (reference=single, no children)			
Married, with children	0.97 (0.58–1.63)	0.95 (0.56–1.62)	1.00 (0.51–1.95)
Single, with children	1.03 (0.57–1.85)	1.00 (0.56–1.76)	1.33 (0.67–2.63)
Married, no children	0.65 (0.39–1.07)	0.64 (0.39–1.05)	**0.42 (0.25**–**0.72)**

*Notes*: *n* = 2906. Data in bold are statistically significant. Model adjusted for: age, sex, ethnicity, highest level of qualification, work status, household income and household composition.

a Model 3 was run only for disabled people (*n* = 955) and was unweighted due to small cell counts.

### Chronicity of food insecurity

Results from multinomial regression analyses examining chronicity food insecurity as the outcome are presented in Supplementary table SA2. Both number and each type of disability were associated with less frequent food insecurity as well as chronic food insecurity. Among disabled people (model 3), however, an increasing number of disabilities was significantly associated with chronic food insecurity (OR: 1.27, 95% CI: 1.09–1.49) but not less frequent food insecurity.

### Sensitivity analysis

Table 3 reports results from sensitivity analyses examining whether associations between disability and food insecurity differ between older and younger adults. We observed stronger associations between number of disabilities, physical disabilities and a combination of physical and mental/cognitive disabilities with food insecurity for younger age groups (defined as <55 and <65), with these relationships becoming non-significant for adults 65+. However, among older adults (55+ or 65+), the odds of food insecurity were particularly high for people with mental/cognitive disabilities, though confidence intervals were large (e.g. OR for mental/cognitive disability 3.36 [95% CI: 1.65–6.87] among <65; OR: 12.6 [1.22–130] for 65+).

**Table 3:** Odds of food insecurity by number and category for adults <55 years of age and 55+ years of age and for adults <65 years of age and 65+ years of age

	Under 55s *n* = 1522		Over 55s *n* = 1348		Under 65s *n* = 2012		Over 65s *n* = 892	
	Model 1^a^	Model 2^a^	Model 1^a^	Model 2^a^	Model 1^b^	Model 2^b^	Model 1^b^	Model 2^b^
	OR (95% CI)	OR (95% CI)	OR (95% CI)	OR (95% CI)	OR (95% CI)	OR (95% CI)	OR (95% CI)	OR (95% CI)
Number								
Continuous	**1.74 (1.44**–**2.10)**	–	**1.45 (1.23**–**1.71)**	**–**	**1.70 (1.46**–**1.97)**	**–**	1.15 (0.92–1.43)	**–**
Category (reference=none)		–		–		–		
Physical only	–	**2.85 (1.35**–**6.02)**	**–**	**2.38 (1.08**–**5.24)**	**–**	**2.34 (1.23**–**4.47)**	**–**	2.97 (0.86–10.2)
Mental/cognitive only	–	**3.10 (1.49**–**6.46)**	**–**	**6.59 (1.36**–**32.1)**	**–**	**3.36 (1.65**–**6.87)**	**–**	**12.6 (1.22**–**130)**
Physical and mental/cognitive	–	**7.32 (2.81**–**19.1)**	**–**	**5.23 (2.42**–**11.3)**	**–**	**7.23 (3.50**–**15.0)**	**–**	1.98 (0.52–7.47)

*Note*: Data in bold are statistically significant.

a Less than 55 and >55 adjusted: age, sex, ethnicity, highest level of qualification, work status, household income and household composition.

b Less than 65 adjusted for age, sex, ethnicity, highest level of qualification, work status, household income and household composition. Greater than 65 adjusted for sex, ethnicity, highest level of qualification, household income and household composition.

## Discussion

This study adds to the current literature by examining how number and type of disabilities are associated with food insecurity. Our results suggest that physical and mental/cognitive disabilities are differentially associated with food insecurity, with a combination of mental and physical disabilities conferring particularly high risk. We also observed that each additional disability conferred higher risk of food insecurity, even conditional on having any disability. These associations were generally stronger for working-age people.

Our findings shed new light on the relationship between disability and food insecurity. We expected different types of disability to have different associations with food insecurity, reflecting the fact that underlying mechanisms might differ due to the heterogeneity of disability experience. Though both physical and mental disabilities were associated with food insecurity, a combination of both was more strongly associated with food insecurity, including severe food insecurity and chronic food insecurity. These results suggest different mechanisms may underlie associations between physical disability and mental-cognitive disability with food insecurity and that when combined, food access is particularly compromised.

Similarly, our findings that an increasing number of disabilities was associated with higher risk of food insecurity could reflect increasing barriers to resources important to achieve food security,^9^ including economic stability.^35^ Research has shown that people on low incomes develop coping strategies to try to avoid food insecurity and shopping around for cheaper food and discounts is often a coping mechanism to secure an adequate diet.^36^ However, for some disabled people, this may be more difficult to implement or may not be an option at all.^3^ Such ‘coping mechanisms’ may become more complex, more costly in terms of finance, impact on other areas of life, and less possible to pursue with an increasing number of disabilities. If increasing numbers of disabilities reflect increased barriers, disabled people may experience an intersection of both multiplied and new disadvantages.^29^ Additionally, barriers may become even harder to navigate if someone has a mental/cognitive disability as well as a physical disability, as suggested by the observed association of a combination of categories with severe food insecurity.

Multiple disabilities may also reflect an increased likelihood of experiencing chronic disadvantage, poverty and marginalization. This may also be a particular concern for people who have life-long and work-limiting disability who may experience more discrimination and be less likely to build up long-term social or financial assets.^9^^,^^11^ We observed that relationships between disability and food insecurity were generally weaker at older ages. This may be due to more effective support services designed for pensioners as well as more generous social security programmes. Disability also becomes more prevalent at older age so may be less closely tied to socio-economic disadvantage. We observed a strong relationship between mental/cognitive disability and food insecurity among older people, however. This group may face unique barriers to information and knowledge related to food access and may face more substantial barriers than physically disabled people to accessing support services.^14^^,^^37^ Mentally/cognitively disabled people may experience more long-term disability and therefore reduced opportunities to building and maintaining resources that prevent against risk factors for food insecurity such as a secure and sufficient income, and asset accumulation for older ages.^2^ Similarly, mentally/cognitively disabled people may be at higher risk of lacking strong informal social networks.^38–40^ This may be a particular risk at older ages when social isolation can be more of a concern.

### Strengths and limitations

Our study uses an internationally standardized measure of food security assessed in a nationally representative sample and incorporated measures of the number and type of disabilities. However, several important limitations should be considered. Our study is based on a relatively small sample size, and our results are based on a cross-sectional analysis that only examined associations, rather than causal relationships. Food insecurity is correlated with other forms of social and economic disadvantage, which may confound the relationship with disability. Socio-demographic variables were limited in the dataset. In particular, age was only provided in age brackets. The crude measure of household income available in the dataset meant that it was not possible to equivalize income by household size. However, we note that including controls for the size of the household did not alter our results. In addition, the limited measures of financial hardship also meant that it was not possible to explore whether insufficiency of income explains the relationship between disability and food insecurity, especially as it does not account for the additional costs of living associated with disabilities. Though the biopsychosocial model of disability informed our conceptualization of how disability relates to food insecurity, variables reflecting the social contexts of disabled people were limited in the dataset. Future research would benefit from further exploring the role of social contexts in conceptualizing disability and food insecurity.

Our measurement of food insecurity measures food insecurity as a result of economic affordability and may not account for non-financial barriers that also reduce disabled people’s food access. However, sufficient financial resources can help to overcome other access barriers, e.g. transport, meal preparation, carers, help with shopping. Importantly, it is an internationally agreed, robust, standardized measure. The internal reliability of the food insecurity scale has been examined in other countries but not in this sample.

The measure of disability available in the dataset did not assess impacts of impairments on activities of daily living nor the severity of disability. Nor did our data allow us to test whether more specific disabilities beyond the broad categorizations of physical disabilities and mental/cognitive disabilities relate differently to food insecurity. Some research in the USA has found that functional and sensory disabilities may not relate to food insecurity in the same ways among older adults.^22^ There was also only a general range of cognitive and mental conditions captured; in particular, mental conditions did not distinguish between common mental disorders and severe psychiatric disorders. However, a strength of this measure is that it is in line with the standard ONS harmonization question for impairments.

Given that our findings point to a significant role for the number of disabilities, future research would benefit from understanding more about how disability severity and types relate to food insecurity outcomes.

## Conclusion and implications

Results from this study suggest that number and type of disabilities are associated with higher risk of food insecurity and chronic food insecurity. They also indicate that a combination of mental/cognitive and physical disability is associated with higher risk of severe food insecurity. Policy-makers may thus consider using targeted and tailored policies to reduce barriers to social and financial inclusion of disabled people to reduce food insecurity. For example, improving access to education, adequate and secure incomes, social care, welfare support and health services as well as supporting reduction of stigma and discrimination, may offer possible targets of public policy to address barriers to food security.

## Supplementary data

Supplementary data are available at *EURPUB* online.

## Funding

The study was funded as part of an Economic and Social Research Council (ESRC) Doctoral Studentship (ES/P000703/1). It also represents independent research partly supported by the ESRC Centre for Society and Mental Health at King’s College London (ESRC Reference: ES/S012567/1).

*Conflicts of interest*: None declared.

Key points

- Physical and mental/cognitive disabilities are associated with food insecurity, but in particular, a combination of both is strongly associated with food insecurity and severe food insecurity.
- An increasing number of disabling conditions is associated with an increased risk of food insecurity as well as chronic food insecurity.
- The increased risk of food insecurity among disabled people with multiple disabilities highlights the need for interventions that reduce multiple disadvantages faced by disabled people.

## Supplementary Material

ckac034_Supplementary_Data
