# Supplementary material for: Food insecurity among disabled adults
Source: Eur J Public Health. 2022 May 13;32(4):593–9. doi: 10.1093/eurpub/ckac034 (PMC9341842; doi:10.1093/eurpub/ckac034)
Supplement: ckac034_Supplementary_Data [file ckac034_supplementary_data.zip › ejph-2021-06-om-0756-File006.docx]

### Table A1. Odds of severe food insecurity for number, category, and number if disabled

|  |  | Model 1 | Model 2 | Model 3* |
| --- | --- | --- | --- | --- |
|  |  | OR (95% CI) | OR (95% CI) | OR (95% CI) |
| Number | |  |  |  |
|  | Continuous | **1.71 (1.41-2.06)** |  | **1.31 (1.12-1.52)** |
| Category  (reference=None) | |  |  |  |
|  | Physical only |  | 1.80 (0.89-3.63) |  |
|  | Mental/Cognitive only |  | 2.06 (0.96-4.41) |  |
|  | Physical & Mental/Cognitive |  | **8.97 (3.54-22.7)** |  |
| Age  (reference=45-54) | |  |  |  |
|  | 16-24 | 1.84 (0.76-4.42) | 1.59 (0.65-3.87) | 0.79 (0.19-3.26) |
|  | 25-34 | **2.99 (1.40-6.38)** | **2.83 (1.31-6.14)** | 1.71 (0.68-4.40) |
|  | 35-44 | 1.96 (0.87-4.41) | 1.89 (0.84-4.24) | 1.86 (0.80-4.31) |
|  | 55-64 | 0.67 (0.30-1.47) | 0.75 (0.35-1.59) | 0.75 (0.36-1.60) |
|  | 65-74 | **0.07 (0.02-0.32)** | **0.08 (0.02-0.35)** | **0.09 (0.02-0.48)** |
|  | 75+ | **0.05 (0.01-0.28)** | **0.07 (0.01-0.35)** | **0.06 (0.01-0.35)** |
| Sex  (reference=Male) | |  |  |  |
|  | Female | 1.17 (0.72-1.89) | 1.08 (0.66-1.77) | 1.45 (0.81-2.58) |
| Ethnicity  (reference=White) | |  |  |  |
|  | Other Ethnicity | 1.24 (0.59-2.62) | 1.20 (0.52-2.76) | 1.35 (0.51-3.62) |
| Qualification (reference=Degree) | |  |  |  |
|  | Other | 1.48 (0.72-3.03) | 1.61 (0.78-3.33) | 2.00 (0.82-4.91) |
|  | No | **2.62 (1.12-6.15)** | **2.63 (1.09-6.39)** | 1.90 (0.70-5.18) |
| Work Status  (reference = In Work) | |  |  |  |
|  | Retired | 1.06 (0.33-3.42) | 0.99 (0.35-2.82) | 2.06 (0.56-7.49) |
|  | Unemployed | **3.96 (1.77-8.85)** | **3.76 (1.56-9.04)** | **5.80 (2.48-13.6)** |
|  | Other | 0.60 (0.31-1.15) | 0.72 (0.39-1.34) | 2.05 (0.98-4.27) |
| HH income (reference= 26k-51k) | |  |  |  |
|  | <£10,399 | **5.94 (2.26-15.6)** | **6.13 (2.36-15.9)** | 2.76 (9.45-8.08) |
|  | £10,400 - £25,999 | **4.02 (1.91-8.47)** | **4.11 (1.93-8.76)** | 1.94 (0.72-5.22) |
|  | >£52,000 | **0.41 (0.11-1.54)** | 0.43 (0.11-1.59) | 0.73 (0.16-3.34 |
|  | Missing | 1.84 (0.85-3.96) | 1.84 (0.86-3.96) | 1.43 (0.48-4.24) |
| HH composition  (reference= Single, no kids) | |  |  |  |
|  | Married, with kids | 0.72 (0.37-1.43) | 0.65 (0.33-1.29) | 0.61 (0.25-1.46) |
|  | Single, with kids | 0.75 (0.36-1.57) | 0.68 (0.31-1.45) | 0.70 (0.30-1.63) |
|  | Married, no kids | **0.42 (0.21-0.82)** | **0.40 (0.20-0.79)** | **0.35 (0.17-0.75)** |

Notes: n=2,906. Data in bold are statistically significant. Model adjusted for: age, sex, ethnicity, highest level of qualification, work status, household income, and household composition.

*Model 3 was run only for disabled people (n=955) and did not use survey weights.
