# Supplementary material for: Food insecurity among disabled adults
Source: Eur J Public Health. 2022 May 13;32(4):593–9. doi: 10.1093/eurpub/ckac034 (PMC9341842; doi:10.1093/eurpub/ckac034)
Supplement: ckac034_Supplementary_Data [file ckac034_supplementary_data.zip › ejph-2021-06-om-0756-File007.docx]

Table A2. Relative risk of frequent food insecurity for number, category, and number if disabled

|  |  | Model A1 | | Model A2 |  | Model A3* |  |
| --- | --- | --- | --- | --- | --- | --- | --- |
|  |  | Sometimes | Often | Sometimes | Often | Sometimes | Often |
|  |  | RRR (95% CI) | RRR (95% CI) | RRR (95% CI) | RRR (95% CI) | RRR (95% CI) | RRR (95% CI) |
| Number | |  |  |  |  |  |  |
|  | Continuous | **1.32 (1.19 - 1.47)** | **1.76 (1.48 - 2.09)** |  |  | 1.02 (0.90-1.16) | **1.27 (1.09-1.49)** |
| Category  (reference = None) | |  |  |  |  |  |  |
|  | Physical only |  |  | **1.59 (1.02 - 2.47)** | **3.20 (1.57 - 6.53)** |  |  |
|  | Mental / Cognitive only |  |  | **2.34 (1.22 - 4.47)** | **3.86 (1.78 – 8.38)** |  |  |
|  | Physical & Mental / Cognitive |  |  | **3.31 (1.98 - 5.54)** | **7.98 (3.26 - 19.5)** |  |  |
| Age  (reference = 45-54) | |  |  |  |  |  |  |
|  | 16-24 | 1.26 (0.66 - 2.41) | **3.69 (1.47 - 9.30)** | 1.25 (0.65 - 2.38) | **3.40 (1.34 - 8.62)** | 0.48 (0.15-1.53) | 2.27 (0.66-7.83) |
|  | 25-34 | 1.25 (0.83 - 1.89) | **2.33 (1.13 – 4.80)** | 1.24 (0.81 – 1.90) | **2.22 (1.07 - 4.59)** | 1.39 (0.65-2.97) | 2.39 (0.89-6.43) |
|  | 35-44 | 1.24 (0.79 - 1.94) | **2.42 (1.10 - 5.36)** | 1.22 (0.78 - 1.93) | **2.30 (1.04 - 5.08)** | 0.96 (0.47-1.97) | 2.26 (0.92-5.58) |
|  | 55-64 | 0.64 (0.40 - 1.02) | 0.82 (0.35 – 1.96) | 0.63 (0.39 - 1.01) | 0.88 (0.37 - 2.07) | **0.47 (0.25-0.86)** | 0.60 (0.27-1.34) |
|  | 65-74 | **0.34 (0.17 - 0.69)** | 0.34 (0.10 - 1.10) | **0.34 (0.17 - 0.70)** | 0.36 (0.12 - 1.04) | **0.26 (0.10-0.65)** | 0.47 (0.12-1.91) |
|  | 75+ | **0.32 (0.14 - 0.76)** | **0.12 (0.02 - 0.64)** | **0.34 (0.15 - 0.78)** | **0.16 (0.03 – 0.75)** | **0.07 (0.02-0.22)** | **0.13 (0.02-0.70)** |
| Sex  (reference = Male) | |  |  |  |  |  |  |
|  | Female | 1.18 (0.88 - 1.59) | **2.06 (1.28 - 3.35)** | 1.18 (0.87 - 1.59) | **1.96 (1.20 - 3.18)** | 1.34 (0.88-2.03) | **1.93 (1.09-3.42)** |
| Ethnicity  (reference = white) | |  |  |  |  |  |  |
|  | Other ethnic group | **1.69 (1.10 - 2.61)** | 1.64 (0.80 - 3.33) | **1.75 (1.12 - 2.72)** | 1.73 (0.82 – 3.65) | **4.82 (2.13-10.87)** | **5.22 (1.92-14.23)** |
| Qualification  (reference = Degree) | |  |  |  |  |  |  |
|  | Other qualification | **1.82 (1.23 - 2.68)** | **2.25 (1.13 - 4.48)** | **1.83 (1.24 - 2.70)** | **2.33 (1.15 - 4.71)** | 1.19 (0.69-2.07) | 2.22 (0.93-5.27) |
|  | No qualifications | **4.20 (2.51 – 7.01)** | **4.30 (1.82 – 10.2)** | **4.28 (2.56 – 7.17)** | **4.30 (1.78 – 10.4)** | **1.95 (1.05-3.65)** | **2.98 (1.14-7.79)** |
| Work Status  (reference = In Work) | |  |  |  |  |  |  |
|  | Retired | **0.43 (0.24 - 0.79)** | 0.82 (0.31 - 2.20) | **0.44 (0.24 - 0.79)** | 0.79 (0.32 - 1.97) | 1.26 (0.55-2.91) | 0.87 (0.23-3.28) |
|  | Unemployed | 1.44 (0.72 - 2.90) | **3.71 (1.87 - 7.36)** | 1.36 (0.68 - 2.73) | **3.52 (1.74 - 7.14)** | 1.92 (0.84-4.38) | **5.46 (2.18-13.7)** |
|  | Other | 0.63 (0.39 - 1.02) | 0.73 (0.40 - 1.32) | 0.63 (0.39 - 1.02) | 0.81 (0.44 - 1.47) | 1.42 (0.82-2.48) | **2.06 (1.01-4.20)** |
| HH Income  (reference= £26,000-51,999) | |  |  |  |  |  |  |
|  | <£10,399 | **2.24 (1.31 - 3.82)** | **2.66 (1.35 - 5.23)** | **2.25 (1.33 - 3.82)** | **2.66 (1.37 - 5.17)** | **2.38 (1.13-5.04)** | **2.76 (1.09-7.01)** |
|  | £10,400 - £25,999 | **1.96 (1.31 - 2.94)** | 1.42 (0.80 - 2.51) | **1.93 (1.28 - 2.90)** | 1.39 (0.80 - 2.41) | 1.79 (0.97-3.33) | 1.51 (0.66-3.45) |
|  | >£52,000 | **0.36 (0.20 - 0.63)** | **0.22 (0.08 - 0.60)** | **0.35 (0.20 - 0.63)** | **0.22 (0.08 - 0.56)** | 0.54 (0.22-1.31) | 0.46 (0.12-1.67) |
|  | Missing | 0.72 (0.46 – 1.11) | **0.47 (0.24 – 0.92)** | 0.72 (0.46 – 1.11) | **0.48 (0.24 – 0.94)** | 1.22 (0.61-2.44) | 0.64 (0.24-1.73) |
| HH Composition  (reference = Single, no children) | |  |  |  |  |  |  |
|  | Married, with children | 1.33 (0.90 - 1.96) | 1.59 (0.80 - 3.17) | 1.34 (0.91 - 1.97) | 1.51 (0.76 - 3.00) | 1.01 (0.51-1.99) | 0.54 (0.21-1.36) |
|  | Single, with children | **1.88 (1.08 - 3.27)** | 1.11 (0.53 - 2.30) | **1.86 (1.07 – 3.22)** | 1.01 (0.51 – 1.99) | 1.78 (0.84-3.76) | 1.33 (0.55-3.22) |
|  | Married, no children | 0.90 (0.62 - 1.32) | 0.96 (0.53 - 1.76) | 0.91 (0.62 – 1.32) | 0.92 (0.51 - 1.66) | **0.46 (0.28-0.75)** | 0.55 (0.28-1.06) |

Notes: n= 2,898. Data in bold are statistically significant. Model adjusted for: age, sex, ethnicity, highest level of qualification, work status, household income, and household composition.

*Model A3 was run only for disabled people (n=951) and was unweighted due to small cell counts.
