# Supplementary material for: Food insecurity among disabled adults
Source: Eur J Public Health. 2022 May 13;32(4):593–9. doi: 10.1093/eurpub/ckac034 (PMC9341842; doi:10.1093/eurpub/ckac034)
Supplement: ckac034_Supplementary_Data [file ckac034_supplementary_data.zip › ejph-2021-06-om-0756-File008.docx]

## Figure A1 Predicted Probability of Severe Food Insecurity by Number of Disabilities and Category


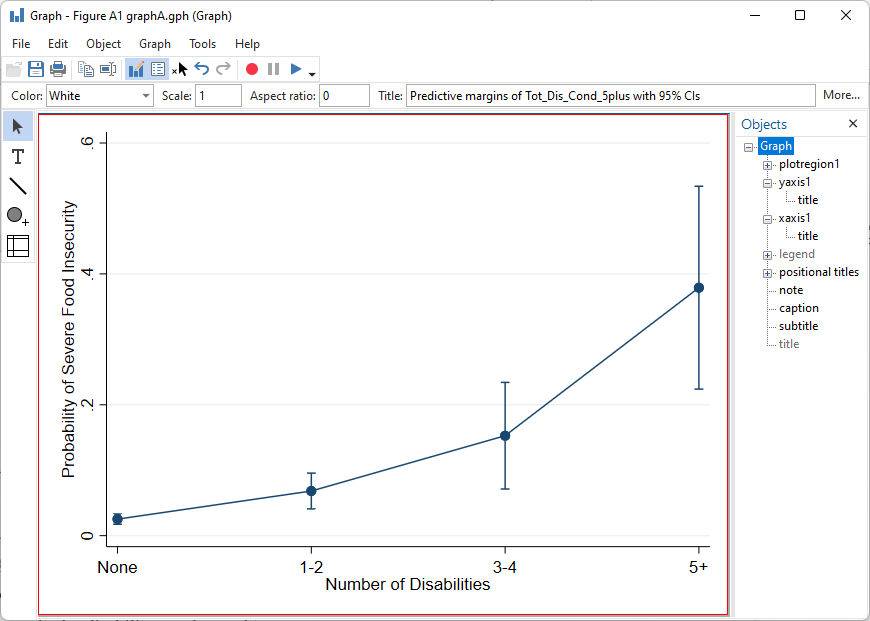

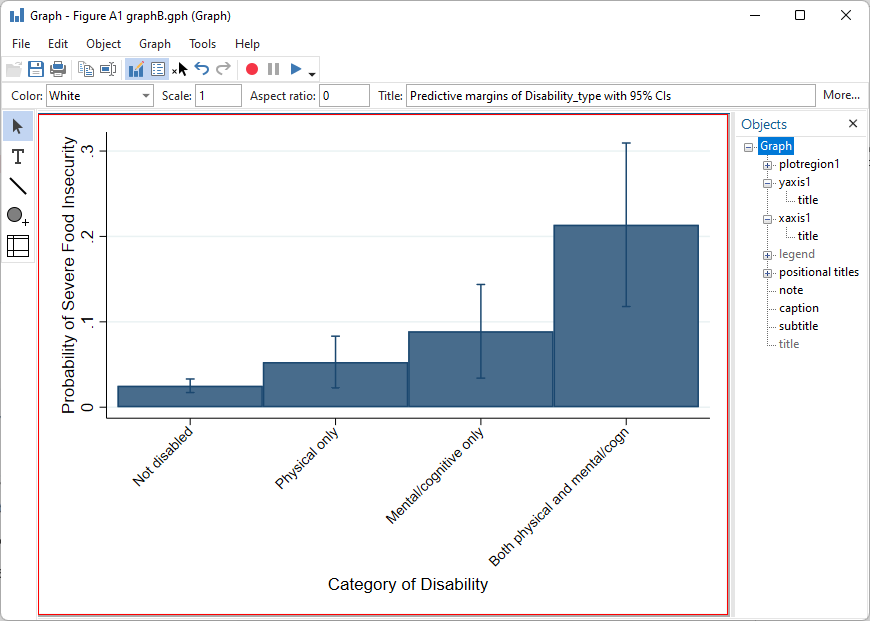


Note: Predicted probabilities adjusted for age

Legend: Probability of severe food insecurity by disability number and category

## Figure A2 Predicted Probability of Chronic Food Insecurity by Number of Disabilities and Category (adjusted for age only)


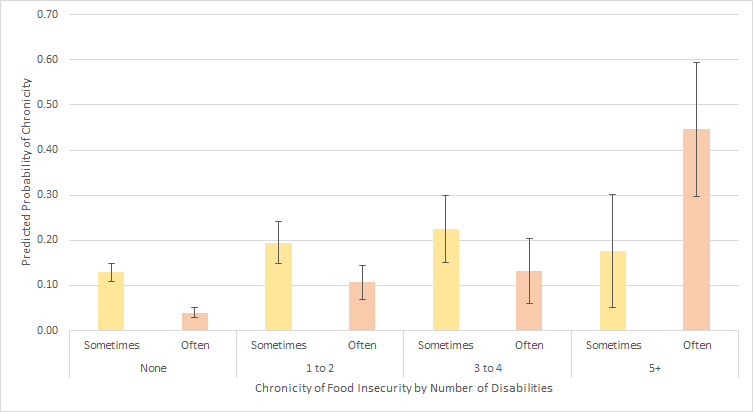


Note: Predicted probabilities adjusted for age

Legend: Probability of chronicity of insecurity by disability number and category
